# Seven core qualities of good vs. bad play? A principal component analysis of 504 children’s play memories and development of a Play Qualities Inventory

**DOI:** 10.3389/fpsyg.2026.1690952

**Published:** 2026-03-27

**Authors:** Andreas Lieberoth, Pernille Strand, Astrid Lehrmann, Helle Marie Skovbjerg, Hanne Hede Jørgensen, Jens-Ole Jensen, Janne Hedegaard Hansen, Anne-Lene Sand, Andreas Roepstorff

**Affiliations:** 1 Danish School of Education (DPU), Aarhus University, Aarhus, Denmark; 2 Kolding School of Design, Kolding, Denmark; 3 Via University College, Aarhus, Denmark; 4 Center for Better Childhood, University College Copenhagen, Copenhagen, Denmark; 5 Aarhus Institute of Advanced Studies (AIAS), Aarhus University, Aarhus, Denmark

**Keywords:** after school programs, child perspectives, factor analysis, psychometrics, primary school, Play Qualities Inventory (PCI), recess

## Abstract

**Introduction:**

This study presents two factor structures suggesting that the qualities and features that children ascribe to subjectively good and bad play situations can be broken down to a relatively low number of central factors. Attempts to model or quantify play have often focused on behaviors, developmental abilities, and pedagogical functions, rather than situational characteristics seen from the child’s perspective. Qualitative studies focused on children’s experiences, on the other hand, often fail to draw patterns across large numbers of children and situations.

**Methods:**

504 primary school students were recalled a recent good or bad play situation, and were asked to match these with short sample statements detailing similar experiences previously collected using episodic interviews with 104 individuals in the age group. Custers of related situational features were then extracted using principal component analysis (PCA).

**Results:**

Clusters consisting of 22 and 7 unique dimensions were identified in the dataset. While some emerging qualities of play like ‘being silly’ or ‘keeping boundaries’ appear uniquely related to discrete activities and preferences, more general factors including ‘social unity’, having ‘a role to play’, being ‘allowed in’, and feeling equipped to participate ‘well enough’, emerge as highly stable dimensions defining good or bad play experiences for children across grades, schools and types of play.

**Discussion:**

and practical application: Findings were combined to propose a seven-dimensional ‘Play Qualities Inventory’ (PQI) for self-report by children ages five to eleven, while the broader backdrop of smaller unique factors provide a new and grounded understanding for discussing the multidimensionality of ‘good’ versus ‘bad’ play as seen through the eyes of children.

## 1 Introduction

This manuscript details the quantitative results of a mixed methods investigation seeking to explore the span of subjective and practical components, that underly children’s perceptions of what constitutes positive and negative play situations. Departing in narrative interviews (Strand et al., 2022; Lieberoth et al., 2025), the present analysis explored which factors can be identified as broadly applicable to a range of real-world play situations, when children are asked to consider ‘good’ or ‘bad’ experiences from memory. A secondary goal was to develop and initially validate a set of self-report scales for children aged 5–11 (PQI), using the words of children to capture the presence or absence of play qualities identified, e.g., on school days.

The 21st century has seen a mounting political and practical interest in children’s play both inside and outside of school. A 2013 report from the UN Child Convention expressed concern that children seem to have fewer opportunities to exercise their right to play, due to ‘child labor, domestic work and increased educational demands.’ Some voices thus view play as something fragile that must be rediscovered and protected (Bishop, 2013), whereas others focus on playing as a resource that should be strategically cultivated (Fisher et al., 2008; Rothlein and Brett, 1987). Many views strive to optimize childhood through play, sports, and academic activities to ensure well-being and development (Shiakou and Belsky, 2013; Watchman and Spencer-Cavaliere, 2017). Whether instrumental or more child-centered, adult beliefs about ‘the good childhood’ are inexorably linked to views of good play.

But what factors delineate good or bad play, if we ask the children themselves, and acknowledge the often ambiguous (Sutton-Smith, 1997) multidimensionality of play? Play is not static and may involve many shifts and permutations over time. Not all play experiences are considered good or even pleasant by children faced with problems like social discord or practical barriers to participation.

Existing models used to document and evaluate play are, however, predominantly meant for adult deployment, and based on adults’ theories of the phenomenological or instrumental qualities of play (Chazan and Chazan, 2000; Knox, 1974; Stagnitti and Unsworth, 2004). According to the American Educational Research Association (1985), however, expert panelists involved in developing psychometric tools should be chosen based on their training, experience, and qualifications. In this light, there are no more qualified experts than children.

As a prelude to the present study, we therefore interviewed 104 children about ‘good’ and ‘bad’ play experiences [previously reported in Strand et al. (2022) and Lieberoth et al. (2025)]. Based on themes identified within their stories, we surveyed a larger number of children aged 5–11 about situational features and feelings in a recent memory of ‘good’ or ‘bad’ play. On the assumption that play is multidimensional, we used principal component analysis to extract two factor models with different benefits: One identifying as many separate components as possible across the multiplicity of play situations participating students had recalled, and one allowing dimension reduction into fewer ‘families’ of related statements that might illustrate recurring core dimensions. Finally, combined these items into the proposed Play Qualities Inventory (PQI) scales, and validated them against their prevalence in ‘good’ versus ‘bad’ play stories. The IPQ is intended as a tool allowing practitioners to assess the presence (or absence) of the seven central play dimensions identified in the present research.

## 2 Previous attempts at quantifying play quality and qualities

The present exploratory study was inspired by how psychometric development seeks dimension reduction in complex psychological areas. Numerous projects have attempted to quantify play, including at the levels of behaviors, traits and attitudes (Kielhofner and Barris, 1984). This is, however linked to a series of challenges, and has often departed in adult rather than child perspectives. The presence of an adult interviewer or observer may affect the children’s reactions or responses. Stanton-Chapman and Schmidt (2021) found that children were reluctant to describe play experiences with other kids, and instead manly focused on parents or other adult figures during interviews. As Bodrova et al. (2023) point out, the lack of good play measurements may lead to inaccurate understandings of play, where observed play situations are more adult-guided than child-controlled. In a recent study on children’s (8 years) play experiences during child-controlled doll play, children started to test the participating adults’ play skills, e.g., by using ‘bad’ words and statements followed by laughter (Jørgensen et al., 2025). Findings of this nature points to the ambiguity of going-bad-while-laughing as an indicator for good play flow from a child perspective, emphasizing children’s ability to make free and limitless choices as one play ‘quality’ (Howard and McInnes, 2010; Jørgensen et al., 2025). Indeed, children do not always experience adult controlled play, games and activities as genuine play, but school-work (Howard and McInnes, 2010). Observations and interview methods are therefore likely to be insufficient when trying to make nuanced statements about the characteristics and quality of play, although they may be central to developing theories and describing patterns (Bodrova et al., 2023).

### 2.1 Observation instruments

Checklists or observation guides based on prevailing theories are commonly scored against standards (Chazan and Chazan, 2000; Knox, 1974; Stagnitti and Unsworth, 2004). For instance, the norm-referenced Child-Initiated Pretend Play Assessment (ChIPPA) (Stagnitti, 2020; Stagnitti and Unsworth, 2004) relies on observing a 2–7 year old child’s interactions with toys and materials to assess the nature of play over a 15-min session: Playing that a doll is sick is counted as an instance of imaginative play, while using a block as a telephone is counted as symbolic play. Similarly, the Children’s Developmental Play Instrument (CDPI) (Chazan and Chazan, 2000) proposes a segmentation of play to describe affective, cognitive, narrative, and developmental components to codify adaptive, conflicted, impulsive, or disorganized play styles. Similarly, the Affect in Play Scale (APS/APS-P) uses a five-minute videotaped play task to code affective and cognitive dimensions (Delvecchio et al., 2016; Fehr and Russ, 2014). More qualitative observation schemes (Westby, 2000) and interview protocols have also been suggested; for instance, relying on conversation during play situations as a window for diagnostic work (Marans et al., 1991) or combining observations of play with adult-guided interviews (Stanton-Chapman and Schmidt, 2021). While anchored in observations, rating instruments are based on academic theories about failings, benefits, and forms of play, for example, relying on notions of play types, stage theory, or play therapy, often to diagnose cognitive or social difficulties (e.g., Gitlin-Weiner et al., 2000). As a result, discussions on ‘good’ and ‘bad’ play mostly refer to benefits or deficiencies that adults detect on the basis of play theories that are occupied with children’s outcomes.

### 2.2 Survey instruments and factor analytic approaches to play

Self-report instruments on play, gaming or playfulness exist for teens or older adults (e.g., Dodd et al., 2021; Trevlas et al., 2003; Shen et al., 2014; Sherry et al., 2006; Staempfli, 2007). Because children are still developing executive functions, linguistic skills, and memory, surveys for younger age groups are rarer, and it has been recommended that questions be vetted thoroughly, for example, by using an expert panel or in-depth cognitive interviewing (Carson, 2007). This can involve children, or professionals speaking from theory or practical experience. Often, the developmental process is, however, opaque. For instance, Dodd and colleagues describe the development of their observational Children’s Play Scale ‘through informal discussion with experts in children’s play from a range of backgrounds including academic, policy and playwork, and by reviewing other relevant measures of children’s time use, physical activity and play’ (Dodd et al., 2021 p 3).

While some instruments are more theoretical, others have been developed using grounded multimethod factor analytical approaches, e.g., to identify uses and gratifications in video games (Sherry et al., 2006) and design scales for adult playfulness at the trait level (Shen et al., 2014). Often children are involved later in the process, e.g., split into calibration and validation groups for EFA (e.g., Trevlas et al., 2003), but one mixed methods study involved adolescents early in searching for predictors of adolescent mental health including playfulness: Staempfli (2007) used a survey partially based on statements collected from 12 students aged 13–19, vetted it for face validity by an adult panel, and tested it with 60 adolescents to identify five factors, which were then collected into a single scale with 20 items dubbed the Adolescent Playfulness Scale (APF20). Clusters of items identified by such procedures can be used as (sub)scales in trait or state measures, or in descriptions and evaluations of circumstances for play, as intended in the combined exploratory and scale development outcomes of the present study. While instruments are thus tested or validated on the age group, the recommendation to use expert panels in psychometric development (Koller et al., 2017; Rust and Golombok, 1999; Zamanzadeh et al., 2014) thus rarely extends to include children’s views and experiences, and children are typically absent from early stages of exploration and inquiry.

### 2.3 Known issues with surveys for children

Filling in a survey requires reading (sometimes aloud by someone else, as in the present study), a certain level of executive function, and a modicum abstract thinking about one’s life, preferences, and experiences. Typical rating scales about children are therefore designed to be filled in by adults to enhance quality compared to self-administration (Touchèque et al., 2016). This shifts to an outside view but enables use with younger children. For instance, the Cerebral Palsy Quality of Life Questionnaire for Children (CPQoL-Child) has two versions: a proxy caregiver version for children aged 4–12 years, and a self-reported measure on children aged 9–12 years (Braccialli et al., 2016). Similarly, the Strengths and Difficulties Questionnaire (SDQ) assessing conduct problems, hyperactivity inattention, emotional symptoms, peer problems, and prosocial behaviour from 4 to 16 years, and comes in a version for the parent, child and teacher which are compared in the final assessment (Goodman, 1997; Mellor and Stokes, 2007). However, a number of questionnaires and self-report instruments for children do also exist. Many were developed for clinical uses including assessment of OCD (Rosa-Alcázar et al., 2014; Wolters et al., 2011), patient quality of life (Braccialli et al., 2016), or difficulties in school (e.g., Goodman, 1997; Mellor and Stokes, 2007). Others address everyday phenomena like motivations (Lieberoth, 2019; Pannekoek et al., 2014) or broad states such as quality of life (Ravens-Sieberer and Bullinger, 1998). Direct responses are especially needed, when the object of a survey is assessing inner thoughts and experiences. For instance, the Persistent and Intrusive Negative Thoughts Scale (PINTS) (Magson et al., 2019) and Perseverative Thinking Questionnaire (PTQ-C) (Bijttebier et al., 2014) generate item responses related to mental and emotional states that adults cannot access. Common age ranges for self-report tools filled in by children individually fall between 8 and 18 years (e.g., Wolters et al., 2011; Bijttebier et al., 2014), with instrument lengths ranging from 5 to 25 items, but they often require supervision by an adult.

Where both adult and child versions exist, it is common to find discrepancies between children’s responses and those of professionals and caregivers (Harding, 2001). It has been noted that the child’s wish to please the adult can affect responses, and questionnaires administered in groups appear to introduce separate biases. A study of 7- to 12-year-olds noted issues involving; group management, group think, maintaining interest, and desires to alter initial responses to make it seem ‘correct’ while hiding their responses from others (Baxter, 2010). In general, children may lose focus or answer complicated questions without fully understanding their meaning, and some children would rather give a random answer than no answer (Bell, 2009). To avoid tendencies to satisfice, give imprecise answers, or try to finish quickly, researchers have emphasized that the labelling of response options is important, questions must be unambiguous, and surveys must not be too long (Ogan et al., 2013). Furthermore, ‘do not know’ options can be left out if they offer no relevant information (Bell, 2009), and negative formulations can be problematic because they confuse response options and require the child to make backwards inferences (Carson, 2007; Bell, 2009). Verbal labels, rather than numerical response options and visual images (e.g., smiley faces), seem to enhance response quality (Bell, 2009). For instance, the Children’s Perceived Locus of Causality Scale (c-PLOK) uses images of pie-charts filled up from one quarter (1), half (2), and three quarters (3) to full (4) for its agree to disagree Likert scale (Lieberoth, 2019; Pannekoek et al., 2014). Similarly, in the Quality of Life Systematic Inventory for Children (QLSI-C) children indicate a walker (very slowly), a cyclist (slowly), a car (quickly) and a plane (very quickly) (Touchèque et al., 2016). The response mode may also affect the results. Tests of a modified QLSI-Q online suggest that tablets allow an ‘interview with technology’ somewhat comparable to adult administration (Touchèque et al., 2016), where children can easily change their answer and go back and forth in questions, thus calibrating themselves to the question format. A study that measured the impact of gamification on a long 79-item web-surveys for children and adolescents from 7 to 15 years found that fewer children requested help using the gamified survey and fewer children used middle responses in both the gamified version and the visually enhanced survey, where the completion time was also longer than that of a pure text version (Mavletova, 2015).

The research to date points to both promises and challenges in distinguishing qualities of play though children’s self-reports. Multimethod factor analytical approaches have shown promise, but the voices of adolescents and children are rare, and often involved only at later stages.

### 2.4 Research goal and background

The present study was initiated as part of an overarching research project aimed at helping adults design better and more inclusive play opportunities for pupils in the 0–3rd grade during breaks and after-school offerings (Sand et al., 2023). While there has been a substantial momentum towards basing pedagogies and views of childhood circumstances on ‘children’s voices’, we found a lack of appropriate instruments for assessing the outcomes in terms of creating ‘good’ play situations at scale. While qualitative and observational data abound, these have rarely if ever been scaled up to test how such isolated insights fit across a large numbers of different children’s experiences. Instead, we found that quantifiable work and instruments largely departed in theories with an instrumental or developmental perspective on the nature and attributes of play (Strand et al., 2022).

The American Educational Research Association (1985) and psychometrics textbooks (Rust and Golombok, 1999) suggests that the development of any psychometric scale should ensure involvement of experts in the field, and depart in the words of individuals with first-hand familiarity with the experiences and processes addressed. For this reason, our objective shifted from assessing effects with off-the-shelf measurements, to developing a “bottom up” model of what should be addressed, when adults wish to understand if a specific play situation, or the play opportunities on offer in, e.g., after-school programs, contain qualities that children themselves would recognize as “good” play. Bu asking about good and bad situations alike, we hoped to identify not just the presence of ‘good’ elements, but also their absence, as well as features that might pose a barrier to participation and enjoyment. As such, our question became: When moving beyond the confines of smaller qualitative studies, what *do* larger groups of children, thinking of a varied body of play situations, recognize as relevant to a good (or bad) play experience?

In light of this overall question, we designed an exploratory study, wishing to depart in children as ‘expert voices’, rather than using ‘off the shelf’ measure of play quality. While our secondary goal was to develop a set of scales that might serve the original purpose of measuring the presence of qualities in ‘good’ play across many children’s play experiences, we did not know *a priori* what these core factors might be. The research goal was thus to identify any sets of ‘core qualities’ of play, that were reliably recognizable as either applicable or non-applicable to a range of play situations, from the quiet to the riotous.

In order to explore the multi-faceted space that is children’s play opportunities and preferences, we adapted phrases from 81 children’s narrative explanations, collected for the purpose of finding examples of what had made recent play situations ‘good’ or ‘bad’. This paper details our testing of these sample statements as agree-disagree self-report items, that might apply to a wider variety of play experiences, and our analysis of how these might be clustered together to reveal more general ‘core’ qualities. For this purpose we generated and scrutinized two factor structures in the resulting dataset, encapsulating 22 factors using extraction based on Kaiser’s criterion to generate as diverse a set of statistical components as possible, and 7 factors using parallel extraction to yield a lower number of components with more statements loading onto each.

The research questions are thus: RQ1: What components can be distinguished? RQ2: Can these be reduced to meaningful clusters of statements, which can be labeled and used to identify ‘core’ qualities (either in terms of feelings or features) that apply to all (or certain categories) of play situations? The secondary objective was to develop potentially useful self-report scales from this factor model, based directly on the words and experiences of children.

## 3 Methods

To ensure children’s voice in all phases, we employed a sequential mixed-methods design inspired by open coding and psychometric development procedures.

In the *sample statement phase* (Section 3.1-2), we generated a corpus of *in vivo* child statements from the appropriate age group. Collection of a varied corpus of episodic child interviews about good or bad play situations has been reported elsewhere (Strand et al., 2022). In preparation for the present analysis, we translated participating children’s stories into a finite number of more generalizable statements (Section 3.2), by identifying central issues, and phrasings with a high degree of content validity (Germain, 2006) as explanations of what had made experiences examples of ‘good’ or ‘bad’ play.

In the *survey phase* (Section 3.3-4), statements selected from the qualitative corpus were developed into a questionnaire and piloted on 81 children. After minor revisions, the resulting 83 item survey was completed with an additional 423 children aged 5–11, for a total of 504 completed responses. Quantitative data was collected using a 1:1 adult-guided survey deployed in schools, where assistants supported children by reading items aloud the iPads used, and clarifying wording when necessary. The children were then asked to agree or disagree with a selection of experiential and descriptive statements while recalling a specific ‘good’ or ‘bad’ play memory.

We conducted two types of factor rotation using principal component analysis (PCA) to identify latent variables connecting children’s experiences through the 81 statements. This stepwise process simultaneously generates building blocks, in the form of recognizable and generally applicable statements, e.g., form expert panelists or though qualitative work with the target audience, and then aids interpretation of the relationships between different markers (such as sentiments expressing how play becomes boring or breaks down), by collecting them into interpretable components through statistical rotation of factor axes (Yaremko et al., 1986) in a process known broadly as dimension reduction (Abdi and Williams, 1985).

Firstly, we extracted all unique dimensions according to Kaiser’s criterion (1960) (eigenvalues >1), disregarding the number of high-loading items. This allowed us to identify the broadest possible palette of elements (sometimes just one or two statements), that explained some variance within our dataset of good or bad play situations. Secondly, to generate a more concise and general set of dimensions that might translate into self-report scales, we extracted factors based on parallel analysis, defined as those with eigenvalues larger than what can be generated from random correlation matrices repeatedly re-sampled from the dataset (Horn, 1965). Bartlett’s test of sphericity *χ*^2^ (3403) = 12,080, *p* < 0.001 confirmed appropriate inter-item correlations for dimension reduction for both extraction modes. Full loading matrices displaying individual items are found in Appendices A, B.

The goal of techniques like PCA is ‘identification of the basic structuring of variables into theoretically meaningful subdimensions’ (Kim and Mueller, 1978, p. 50). Thus, once factors and their internal validity have been determined through rotation, an interpretative process commences to assign that ‘meaning’ and ensure content validity though systematic conceptual evaluation and ‘naming’ (e.g., Shen et al., 2014). After factors were extracted, an expert panel with background in play and cultural studies, was convened to interpret common threads in each cluster of items. Naming lends theoretical meaning to the statistical patterns, hints at the underlying latent variable, and further enhances content validity by pointing out items that seem central or more peripheral.

Based on this, we proposed a set of 7 reduced self-report scales, based on the dimensions that explained most variance within the dataset (Section 7). In order to validated these, we conducted Confirmatory Factor Analyses and evaluate the precision of each subscale for identifying good versus bad play stories within the dataset.

### 3.1 Participants

Children were recruited from four suburban schools, two of which took part in a four-year research project (Sand et al., 2023), and selected to represent both ends of the socioeconomic spectrum. Informed consent was collected from caregivers, and information as well as the opportunity to opt out were reiterated to all students in the participating years before each child interview. Data collection included no personal or biomedical data and therefore fell outside further purview of the regional ethics review board. The study was conducted in accordance with national and institutional ethical guidelines for research involving minors, and research in schools.

In the qualitative phase, 104 first- and second-grade students were interviewed. Of these, one student opted out midway. Demographic data were not collected in this stage to preserve anonymity in accordance with standards for school research. Studies of saturation in bottom-up research such as grounded theory suggests that recurrence and coverage of relatively common sentiments can be achieved after 9–22 interviews (Saunders et al., 2018; Hennink and Kaiser, 2022), but given our focus on both ‘good’ and ‘bad’ situations, and the varied forms play can take, we aimed for at least five times this number of play stories for qualitative our corpus.

Samples of 300, or at least 5–10 participants per variable have been recommended for principal component analysis (Field, 2013). We thus aimed for a minimum of 300 children, while hoping to approach 435 to satisfy both lower bounds. The final sample size of 504 was determined by the number of children available from the four schools (see Table 1).

**Table 1:** Descriptive statistics for age, grade and gender across the two study phases.

Phase	Age	Grade	Gender
*Phase 1: Qualitative interview data*			
Interviews (*N* = 104)	Not specified	1 + 2 grade	Not specified
*Phase 2: Qualitative questionnaire data*			
Survey pilot (*N* = 81)	6–11	0 grade: 11	Female: 44
	M = 8.35	1 grade: 22	Male: 37
	SD = 1.12	2 grade: 16	
		3 grade: 22	
Survey (*N* = 423)	5–11	0 grade: 105	Male: 218
	M = 7.68	1 grade: 86	Male: 205
	SD = 1.19	2 grade: 122	
		3 grade: 110	
Total (*N* = 504)	5–11	0 grade:116	Female: 262
	M = 7.79	1 grade: 108	Male: 242
	SD = 1.20	2 grade: 148	
		3 grade: 132	

### 3.2 Sample statement pool selection

In order to generate a pool of age-valid sample statements before the quantitative phase commenced, we interviewed a panel of 104 ‘child experts’ on play experiences from their day-to-day lives. This process has been detailed elsewhere (Strand et al., 2022). Children were interviewed in pairs or trios at their schools, and asked to each recall one play situation, alternating between ‘good’ or ‘bad play.’ Inspired by the episodic interview method (Flick, 2000), the children were encouraged to briefly re-experience the situation in their minds. We then asked what made the play situation good or bad, and prompted examples of feelings and details about what had led to the best moments or problems like breakdowns or barriers to participation.

Children often used descriptive statements (e.g., ‘it was the kind of game where you could win!’) to call attention to salient aspects, whereas eliciting thoughts and emotions required more prompting (Strand et al., 2022). Words like ‘fun’ and ‘boring’ were used frequently enough, to become almost meaningless ‘black hole concepts’ (Cole, 2006). After each interview, interviewers thus continually added to a working corpus (Braun and Clarke, 2006) of sample statements by preserving useful *in vivo* expressions (as per Saldaña, 2015).

In order to generate a list of sample statements encompassing a wide variety of themes, we broke each narrative down into its constituent elements using thematic analysis (Giorgi and Giorgi, 2004; Braun and Clarke, 2006). This required acknowledging larger patterns as well as the multidimensionality emerging from a plethora of play situations recounted by children with varying preferences. 108 positive, 123 negative and 9 neutral sample statements were selected as candidates for survey use, representing items that both described inner experiences at a phenomenological level (Flick, 2000; Giorgi and Giorgi, 2004) and descriptive items calling attention to situational features.

We then excluded statements that required a particular element, such as ‘ball play’ but we included experiences about, e.g., ball games that could also apply to other activities, like others ‘getting too rough,’ focus on ‘winning,’ or being ‘good at it.’ For instance, a sentence like *‘It’s good because it’s cozy (‘hyggeligt’), and we are all running around together, and sometimes you’ll forget your number…’* encompassed multiple dimensions like large-scale play, rules, social play, hygge, cognitive challenge, and physical activity. Since statements expressing ‘goofing up’ and playing ‘together’ had already been found, this fairly dense snippet resulted in two new themes (as per Braun and Clarke, 2006) captured by sample statements: ‘It was cozy’ (hyggeligt) and a rephrasing of the quite specific experience of cognitive challenge to ‘You had to use your head’. *‘We played in the sandbox and made mud pies, and then I made one, and it was just perfect, but then…’* similarly contains very specific information (sandbox) that would not be applicable to most play situations, but ‘it was just perfect’ is an interesting expression of positive valence. ‘We’-phrasings where only retained when the social component was central, to address solitary as well as social play whenever possible. By also removing (near-)duplicate or highly related statements, and statements unlikely to be understood outside their original context, we were able to reduce the corpus to 83 items, phrased in general terms like ‘you could…’ or ‘someone tried to…’

## 4 Quantitative data collection

### 4.1 Materials and procedure

The Qualtrics survey resulting from the sample statement pool (Section 3.2-3) was deployed as one-on-one interviews using iPads at the children’s schools, with assistants reading information, questions, and options to the child.

In accordance with Danish ethical standards for school-based research, assistants read the informed consent statement, checking for understanding, and stressing that the children were allowed to opt out, even if their parents had approved their participation, and that they were allowed to leave at any time.

After supplying basic demographic information, Qualtrics randomly assigned each interview to focus on either a good or bad play experience they had been part of at school from the last couple of weeks. Once the child thought of a play situation, and was able to visualize it in some detail, the interviewer asked them to describe the experience, and wrote a brief description into a free-text field.

The interviewer then told the child ‘Now I am going to read some things, that other kids have told us about their own play experiences. I need you to tell me if these things fit with your play.’ Two clearly positive and negative test items (‘it was fun!’ and ‘I couldn’t be bothered’) were used to calibrate the child. The interviewer helped children who seemed to misunderstand the five-point agree/disagree response scale. Then 83 sample statements were presented. Finally, the assistant made a note on the kind of play described for descriptive purposes (Hughes, 2002; Whitebread et al., 2017; see Table 2).

**Table 2:** Types of play episodes remembered by participants.

Play type	Frequency
Physical	86
Objects play	32
Symbolic play	30
Fantasy	147
Rule based	233

Play types coded according to Whitebread et al. (2017). Play situations can be ambiguous or contain features of more than one type.

### 4.2 Pilot

83 statements were entered into the Qualtrics platform and tested with 81 ‘child experts’ aged 6–11, to ensure face validity and identify content issues (Zamanzadeh et al., 2014). The pilot had seven response options: ‘strongly disagree, disagree, agree, strongly agree, and neither/nor’. In addition, ‘does not fit the play situation’ and ‘item not understood’ options were initially available to the interviewer. The survey was presented on an iPad, and read to the children by assistants, who filled in the responses while taking notes of their reactions and parts of the protocol or items that caused problems or confusion. All items were understood more than 95% of the time, so a full battery of 83 items was carried over to further data collection. As the items used in the pilot all appear in the final survey, the 81 pilot responses were carried forward into the final dataset.

## 5 Data analysis

Recalled memories were 53.08% good and 46.92% bad play experiences. Individual item responses were non-normally distributed, with SDs on the 1–5 Likert Scale ranging between one and two, showing a tendency towards U-shaped distributions, but all options were used occasionally. The mode responses were ‘strongly agree,’ ‘agree,’ or ‘disagree.’ The tendency towards extreme responses among younger respondents is consistent with previous findings (Batchelor and Miao, 2016).

The option ‘doesn’t fit the play situation’ was used 296 times and ‘item not understood’ 241 times. Children were able to use each item in more than 95% of the cases, except for two items (6.34 and 7.99% ‘doesn’t fit the play situation’-responses) to do with creating products. While no set thresholds exist for when to exclude an item based on face validity concerns, these distributions fall well within the recommended ranges (Koller et al., 2017; Zamanzadeh et al., 2014).

## 6 Results

Principal component analyses (PCA) were used to extract clusters of related statements. We describe each emerging component and compare the two factor structures in light of their relationships to positive and negative play memories.

### 6.1 Extraction using Kaiser’s criterion

**Figure 1:**
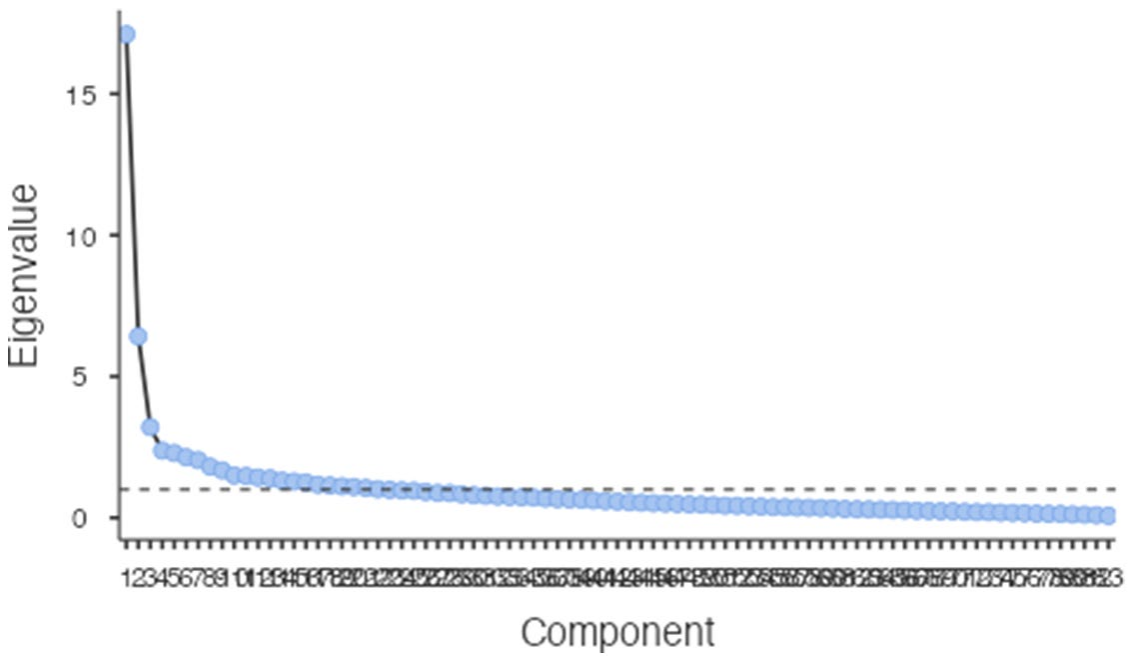
Scree plot, first extraction.

Orthogonal varimax rotation in the R Psych module implemented in the Jamovi environment (Revelle, 2019), with factor extraction based on Kaiser’s criterion (eigenvalues < 0.1) resulted in 22 unique factors with Eigenvalues from 17.11 to 1.01, explaining a cumulative 66.6% of the variance (see Table 3 for individual factors). The Kayser-Meyer-Olkin measure verified the sampling adequacy for the analysis, = 0.88 (‘great’, according to Field, 2013), and KMO values for individual items were < 0.56 keeping above the acceptable minimum of 0.5. The resulting component model accounts for 66.6% of the variance, an included all 83. Each component accounted for between 16.41 and 1.60% of the variance, with many comprising only one or two items (see Figure 1 and Table 1).

The 22 components resulting from this mode of extraction are suitable for a broad exploration of the range of identifiable factors within the dataset, that may be relevant to some children’s play experiences. Their exact relationships are open to interpretation, but we offer summary suggestions in Table 3.

**Table 3:** Component loading matrix, extraction using Kaiser’s criterion.

Factor number	Component designation		SS loadings	% of variance	Cumulative % of variance	Description
1	**The play feeling**	25 items	13.62	16.41	16.4	Component 1 contains expressions of positive versus negative feelings as well as engagement and alignment within the activity. It is by far the largest, mainly due to variations on ‘boo/hooray’ statements.
2	**Alone, excluded**	6 items	3.69	4.45	20.9	Component 2 describes situations involving a predominantly social barrier to participation, through ostracization by others, or an inability to find someone to play with. Here, social pushback clearly carries an element of play crisis for all involved.
3	**Wild and physical**	6 items	3.16	3.80	24.7	Component 3 describes features of bodily action in play, including arousal, wildness, and pushing oneself.
4	**Fantasy and creation**	4 items	2.64	3.18	27.8	Component 4 encapsulates creative activity and relates to the creation of products that can be kept or shown to others. Playing that ‘it was real’ loads on this factor as well, showing an interesting link between fantasy play and creative processes.
5	**Mischief**	3 items	2.31	2.78	30.6	Component 5 encapsulates transgression, including being silly, cheeky, and getting up to no good together.
6	**Risk and transgression**	3 items	2.31	2.78	33.4	Component 6 is ambiguous in semantic valence, describing risk through boundary crossing, getting scared, and goofing up.
7	**Premise misalignment**	3 items	2.13	2.57	36.0	Component 7 describes instances of play misalignment when some are perceived as not playing ‘like you are supposed to,’ refusing to participate, and/or taking it ‘too seriously.’
8	**Inventing and changing play together**	4 items	2.09	2.52	38.5	Component 8 converges on inventing and changing play together. The factor most strongly emerges from a strong loading on ‘the kind of game you can invent together’ with elements of helping each other, making changes along the way, and, most peripherally, being able to ‘use your imagination.’
9	**Developing competences**	3 items	2.05	2.47	41.0	Component 9 has to do with developing mastery in activities where practice makes you better and you can learn something. Slightly more peripherally, ‘you can win,’ hinting at building mastery in sports and games.
10	**Everyone was allowed**	2 items	1.96	2.36	43.3	Component 10 is a two-item component describing the ease of joining in, when ‘everyone is allowed’ to try or join.
11	**Repetition with adult supervision**	2 items	1.92	2.31	45.6	Component 11 encapsulates grind and repetition, with adult involvement. Either adult mandates are somehow antithetical to playfulness, or the need for an adult follows from play breakdowns.
12	**Unreality**	2 items	1.87	2.25	47.9	Component 12 encapsulates the unreal nature of play in two features, where participants can try things that you are not allowed to, or indeed cannot, ‘in real life.’
13	**New people, new experiences**	3 items	1.81	2.18	50.1	Component 13 collects qualities related to novelty and new relationships, with items expressing playing with someone new, and more peripherally, trying something new.
14	**Low agency**	3 items	1.80	2.17	52.2	Component 14 encapsulates low agency and nonparticipation. An item describes when adults steer the game but the child finds it boring. Others are about just looking on or doing nothing in the periphery.
15	**Our play!**	3 items	1.76	2.12	54.4	Component 15 encapsulates play processes that are well known, and played with select cohorts, while others are felt to stand on the outside. This fascinating our play factor illustrates that familiarity and ownership are important qualities in some play experiences, and that exclusion can be a legitimate way of protecting the play order.
16	**Too little time’**	1 item	1.60	1.93	56.3	Components 16, 17, 18 and 20 are all single items which do not load strongly on the above, but may illustrate salient components in various play experiences: ‘There was too little time’, ‘It ended up as/became a game, where everyone just played differently’, ‘it was really noisy’, ‘you had to use your head”
17	**Everyone ended up playing differently’**	1 item	1.60	1.92	58.2	Components 16, 17, 18 and 20 are all single items which do not load strongly on the above
18	**Noisy’**	1 item	1.50	1.81	60.0	Components 16, 17, 18 and 20 are all single items which do not load strongly on the above
19	Relaxation, energy, interruption, scarcity*	4 items	1.42	1.72	61.7	Component 19 is a composite in which the experience of coming to relax and being disturbed loads positively, encapsulating peace versus interruption. Gaining energy from the activity and a scarcity of resources both load negatively, which is difficult to interpret, but perhaps illustrate shifting conditions of arousal and (un)hindered engagement.
20	**Using your head’**	1 item	1.37	1.65	63.4	Components 16, 17, 18 and 20 are all single items which do not load strongly on the above
21	**Difficulty**	2 items	1.33	1.60	65.0	Component 21 encapsulates difficult requirements, and the experience of disrupting play for others.
22	**Making my own decisions’**	1 item	1.33	1.60	66.6	Component 22 encompasses making your own choices, and no one else in charge of your play.

See Appendix B for individual items and loadings.

### 6.2 Extraction using parallel analysis

In order to also generate a more concise set of factors, with a high number of items potentially loading on to the same latent variables, an orthogonal varimax rotation was conducted with factor extraction based on parallel analysis (as per Horn, 1965), resulting in seven unique factors with Eigenvalues l from 17.11 to 2.04, explaining a cumulative 42.9% of the variance (see Table 4). The Kayser-Meyer-Olkin measure verified the sampling adequacy of the 83 items for the analysis, = 0.88 (Field, 2013), and KMO values for individual items were < 0.56, keeping above the acceptable minimum of 0.5. The scree plot illustrates the automatic extraction relative to the simulations. The resulting component model consisting of seven factors reduced dimensionality considerably compared to the first extraction. The component model included 74 items. Components accounted for between 16.44 and 3.38% of the variance.

**Table 4:** Component loading matrix, parallel extraction.

Factor number	Component designation		SS Loadings	% of variance explained	Cumulative% of variance explained	Description
1	**‘Play feeling’**	28 items	13.65	16.44	16.4	A positive ‘play feeling’, encompassing engagement and enjoyment, with feelings such as boredom or sadness loading negatively. As the first factor, it has many high- and low-loading items, that are both positive and negative. In addition, it has distinct subthemes related to playing together and alignment among participants, or a ‘social play feeling’. For instance, children described good play as when “you can laugh,” “it’s really, really good [referring to the play],” or “when you get a smile on your face,” whereas bad play was described as “boring,” “annoying,” or “feeling sad inside.” Strongly indicative of a good play memory (see Table 5)
2	**‘Disharmony and exclusion’**	14 items	4.84	5.83	22.3	Items phrased in a negative way relating to being kept out or not allowed in, e.g., due to shortcomings like ‘goofing up’ or ‘misbehaving,’ or boundary conflicts as children interrupt or try to make their way in. More peripheral items form a backdrop of not being able to find anyone to “be with,” “getting hurt,” or others “taking it too seriously,” suggesting competing dynamics, in which the ‘correct way of playing’ is explicitly felt, especially by those struggling to participate. For example, some children describe exclusion through statements such as “they said I could not join,” or frustration when “someone ruins it.”
3	**‘Imagination and possibility’**	11 items	4.02	4.84	27.1	Simultaneously encompasses getting ideas, doing something you cannot in real life, and imagining that ‘it was real’ as well as the creative production of something to show off or keep More peripheral items include trying or learning something new and paradoxically, both playing by oneself or inventing the play together. A vivid example of the imaginative process was seen in the qualitative work, when two boys play zombie-apocalypse during recess, where survival depends on them defending themselves against terrifying zombies. When the adult asks why this play is so enjoyable, the boys respond: “Because you get to kill someone.” This illustrates how imaginative play allows children to explore scenarios beyond everyday reality.
4	**‘Wild and exciting’**	7 items	3.56	4.29	31.4	Describes the hallmarks of ‘rough’ and ‘tough’ action-packed play. Which is often experienced negatively as hard, noisy, and unrelaxed. Although predominantly negative, this factor also captures the appeal of wild play when it is balanced and goes well, highlighting the tension between risk, excitement, and discomfort.
5	**‘Having something to do’**	9 items	3.51	4.23	35.6	Collects not having ‘Something to do’ and describes passivity, everyone doing their own thing, or feelings of being pressed into boring participation by adults, often due to limited access to materials or not getting to try what made the activity fun. The item loading most strongly was “I got to know someone”, and this component includes playing with someone new.
6	**‘Accessibility, competence and challenge’**	10 items	3.19	3.85	39.5	Describes the activities of a more open and ludic nature, where everyone is allowed to join in and try, placing a focus on practicing and succeeding using one’s body or mind. It reflects the experience of being appropriately challenged and improving through practice. For example, children described these situations as when “everyone could join,” you got to try,” or “you became better at it.”
7	**‘Silliness and transgression’**	4 items	2.81	3.38	42.9	It encompasses elements of mischief and boundary exploration, being zany and cheeky, and getting up to no good together, coupled with the autonomy to make up one’s own play premise. Component 7 only contains four items but explains almost as much variance as the much larger component 6. For example, children described “doing something you are not allowed to,” “being silly,” or “making up your own play,” capturing how playful disobedience can be fun as long as the play is going well.

See Appendix C for individual item loadings.

## 7 Developing a Play Qualities Inventory for children (PQI) from results self-report scales for children

Together, the 22 and 7 components illustrate what features and words children themselves seem to recognize and agree on, when thinking of memories for situations, that they had themselves chosen as recent exemplars either a good or bad play.

In addition to identifying qualities of play as seen through the eyes of children, the study departed in a wish to possibly of evaluate “good play” with scales composed of children’s statements. Following the expert panel procedure (Koller et al., 2017; Rust and Golombok, 1999; Zamanzadeh et al., 2014) a group of evaluators with extensive experience in play research was convened to scrutinize the two factor structures, and aid in generating the shorthand labels for theoretical constructs that reflect possible underlying latent variables in each component. Based on this, the second 7-factor model was translated into a set of psychometric scales dubbed PQI.

The large factor one is a candidate for a unidimensional “good play-scale.” However, the factor contains quite a few high-loading social statements including “everyone had fun together” and “we were good friends”, suggesting that a positive play feeling is intricately related to social alignment.

For this main “good or bad” play scale, we deliberately tried to avoid phrasings like “fun” and “perfect”, in order to make room for a broader range of expressions including coziness (the famous Danish “hygge”), happiness, annoyance and “sadness inside”, which statistics in the 0.8–0.9 range illustrating good coherence with a slightly wider span in component meanings (Rust and Golombok, 1999). The expert panel recommended splitting factor one into two distinct sets of items: Those related to an individual play feeling, and those related to a social play feeling, or removing the seven items with a social component. The remaining factors were reduced into shorter scales based on item loadings, and theoretical the content validity of each item in relation to the shorthand theoretical construct, the panel saw each scale as a reflection of.

There are no clear standards for when an item should be included or excluded in a self-report scale. Since the smallest component of the parallel extraction had four items with factor loadings of 0.32–0.42 and uniquenesses > 0.40, these values were used as lower and upper benchmarks for reduction of the other factors. We aimed for four items per scale for maximum reliability (Peter, 1979; Rust and Golombok, 1999), while matching the minimum number of possible items dictated by the smallest factor and simultaneously avoiding known issues related to validity testing of three-item scales (Czerwiński and Atroszko, 2023). A balance between items with positive and negative loadings (and thus valence direction) was desired, but possible only in the case of the large component 1 (*Play feeling*).

### 7.1 Internal and discriminant validity

Confirmatory factor analysis (CFA) was used to reduce the “core components” from extraction 2 down to 3–5 items per scale (as per Robinson, 2018), with items being selected based on statistical coherence, and theoretical fit with latent constructs identified by panel deliberation (see Table 5 and Appendix A).

**Table 5:** Scale reliability statistics for Play Qualities Inventory and relations to the original seven components extracted with parallel analysis.

	*M*	SD	Cronbach’s α	McDonald’s ω	r original component	r play feeling
Play feeling	3.95	1.11	0.873	0.881	0.902**	–
With social items	3.87	1.09	0.891	0.899	0.910**	0.980**
Inclusion	1.75	0.98	0.707	0.719	0.641**.	−0.541**
With disharmony items	1.92	0.89	0.692	0.708	0.752**	−0.516**
Imagination	3.01	1.12	0.592	0.594	0.810**	0.299**
With creation/performance items	2.76	1.01	0.683	0.688	0.862**	0.348**
Wildness	2.77	1.09	0.616	0.632	0.786**	−0.349**
Something to do	2.10	1.03	0.700	0.708	0.656**	−0.564**
Accessibility	4.06	0.91	0.575	0.612	0.696**	0.513**
Silliness and transgression	3.03	1.09	0.614	0.631	0.800**	0.138**

*p < 0.05, **p < 0.01.

The scales make room for a range of expressions of the shorthand construct, including coziness (‘hygge’) happiness, annoyance and ‘sadness inside’, which all loaded strongly on the “Play feeling” scale. Together the reduced scales proposed here yielded *ω* and *α* values in the 0.6–0.9 range illustrating good coherence (as per Rust and Golombok, 1999) with a slightly wider span in the meaning of items describing, e.g., imagination and accessibility.

In the interest of content- and discriminatory validity, social items were removed from the main ‘Play feeling’ scale, so it would solely be a measure of positive or negative valence, and applicable to all play situations, solo or social. Following the same logic, items relating specifically to creation/performance were removed from the imagination scale, and feelings of social conflict/disharmony from the Inclusion scale. Expanded scales including these supplementary items, can be found in Appendix A. Finally, selected items were chosen to be reverse-coded, allowing the titles to specifically reflect positive rather than negative dimensions of play—i.e. *inclusion* rather than exclusion.

In order to test whether the seven resulting scales gave unique indications of a good play experience, or described more varying but coherent preferences among the children, inter-scale correlations, and the ability of each scale to discriminate between whether childrens’ ratings related to a memory recalled to be *a priori* “good” or “bad.”

Discriminant validity was explored using Krushkal-Wallis ANOVA with Dwass-Steel-Critchlow-Flinger pairwise comparisons. As seen in Table 5 all the scales, except *Transgression,* show high precision in discriminating between play stories about good and bad experiences within the dataset. *Social disharmony*, having *Nothing to do*, and to a lesser degree *Wildness*, are associated with bad play memories. Imagination and wildness fall short of large effect sizes, suggesting variations in child preferences and limited relevance to some play situations.

**Figure 2:**
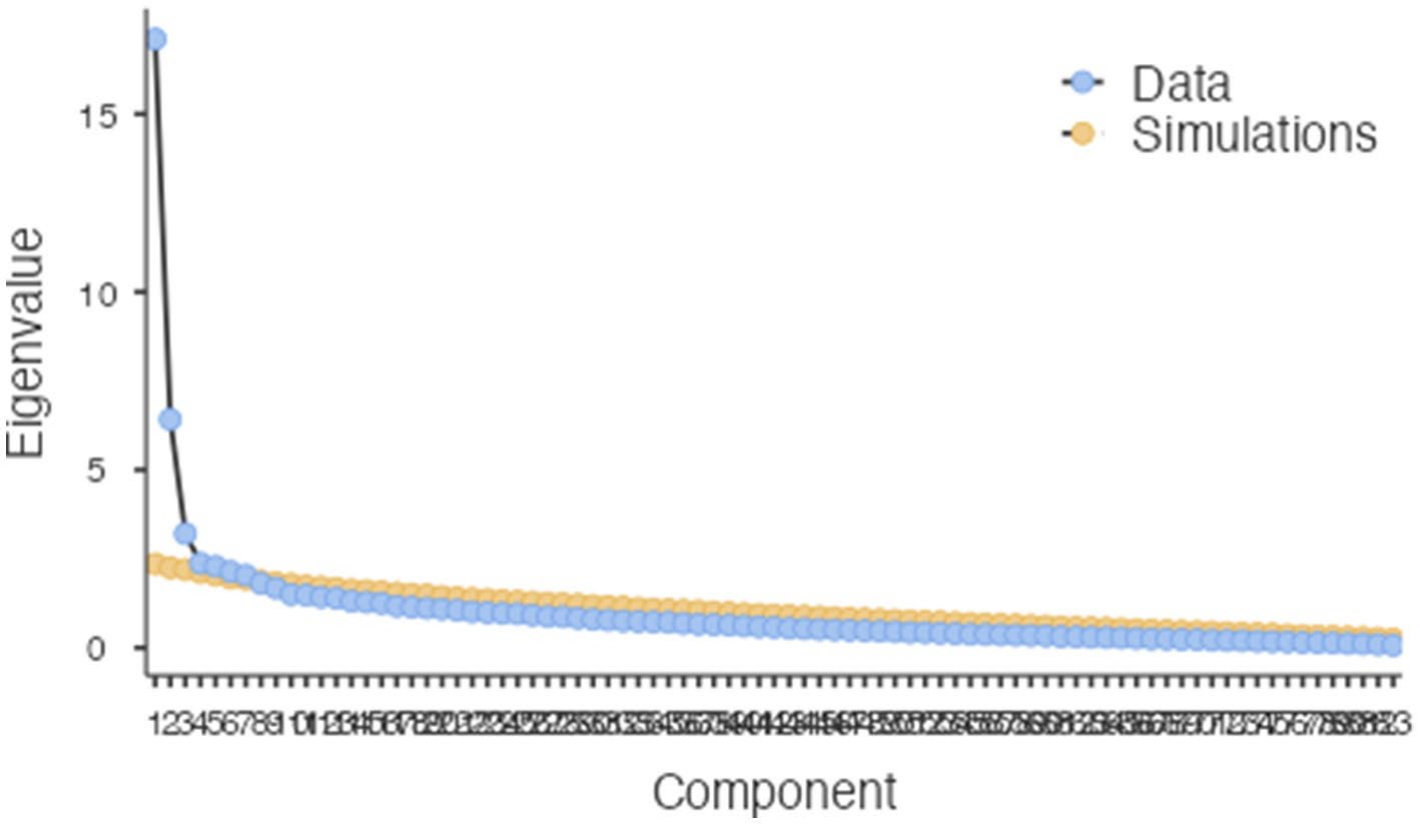
Scree plot, second extraction.

Furthermore, all components correlate either positively or negatively with *Play Feeling* as seen in Figure 2, as well as with *Accessibility*, while the remaining scales do not correlate systematically with each other. This difference, and the varying effect sizes, suggests that play situations dominated by features such as physical activity or imaginative creation, or which involve experiences of transgression, may offer very different opportunities and feelings to different kids. This relationship also indicates that Play Feeling can be used as a parsimonious ‘main scale’ of positive play experiences, if researchers are not interested in the more specific facets.

## 8 Discussion

We set out to identify central dimensions found across good versus bad play experiences. Using children as domain experts in the different steps from interviews to surveys, we identified two factor structures, showing both common patterns across children’s utterances, and more specific issues and preferences. Based on these, we proposed a seven dimension instrument for measuring the theoretically meaningful qualities.

In statistical terms we find that play is extremely multidimensional. Qualitative analyses are excellent for discovering nuances and variations, they might give us a hint of how good and bad play experiences look like in the situation and from outside the child-controlled play. However, the sheer volume of observations needed makes analyses of interviews and fieldwork ill-equipped to detect some patterns, such as co-occurring dimensions within situations spanning different play experiences. PCA is a tool for collapsing a large number of qualities into a smaller set of dimensions, while still maximising the variance that can be understood with the emergent components (Kherif and Latypova, 2020). Applying PCA to qualitative sample statements from individual play stories, allowed us expanded across a much larger array of play memories (i.e., the situation each survey participant thought about when answering), thus leaving a situation- or person-cantered epistemology in favour of a more topographical approach (Godwin et al., 2021).

Scritinizing dimensions confirms that this technique can reduce the high dimensionality of play to a more manageable and practical model. This larger pool of unique “bottom up” factors reveals 22 and 7 relatively independent qualities of play experiences.

We discovered that children describe ‘good’ or ‘bad’ qualities in terms of situational facets, feelings, or both, with emotions often requiring more prompting and social elements prominent at all levels. The results show that the hallmarks of a good ‘play feeling’ consisting of enjoyment, activity and (if playing together) alignment among participants seems almost universal to children. In good play memories it is there, while it is absent in recollections of memories of non-preferred play or where things turn bad.

Each component thus reveals a cluster of elements that are not necessarily or exclusively indicative of good or bad play, but they all reveal elements that seem to group together in children’s understandings, when trying to characterize a play memory. Several relationships within the data can be used to illustrate the relationship between clustered items and good or bad memories: In the first extraction, component 2, 7, 11 and 22 comprises partially of statements that also load negatively on component 1 (The overall positive “play feeling” found in both dimension reduction methods), suggesting that they are somewhat antithetical to this positive or harmonious play experiences. Scrutinizing inter-scale correlations (Table 6) it becomes clear, that the statements encapsulated in social disharmony, physical wildness, and (the lack of) something to do were often, but not exclusively, used to describe situations that were *no*t also described as good, e.g., “fun” or “you could get a smile on your face” but rather, e.g., “boring” and “annoying.” All scales were also found to be usable to distinguish whether a set of statements came from an *a priori* good or bad memory (Table 7), but *something to do*, *accessibility* and *play feeling* displayed the largest effect sizes when discriminating good from bad play memories. As such, “Play Feeling” is by far the best single indicator of whether a play situation *felt* good or bad in terms of describing positively (laugh, good, you can get a smile on your face)vs. negatively (boring, annoying, feeling bad inside) valanced emotions elicited in play. As such, these phrasings could also be useful simply for talking to children about play (c.f. Strand et al., 2022).

**Table 6:** Inter-scale correlations.

	Play feeling	Social disharmony	Imagination	Wildness	Nothing to do	Accessibility	Transgression
Play feeling’							
	—						
Social disharmony	−0.516***	—					
	< 0.001	—					
Imagination	0.299***	−0.011	—				
	< 0.001	0.822	—				
Phys. wildness	−0.349***	0.263***	−0.157**	—			
	< 0.001	< 0.001	0.001	—			
Something to do	−0.564***	0.538***	−0.077	0.079	—		
	< 0.001	< 0.001	0.114	0.101	—		
Accessibility	0.513***	−0.280***	0.245***	−0.142**	−0.315***	—	
	< 0.001	< 0.001	< 0.001	0.003	< 0.001	—	
Transgression and silliness	0.138**	0.061	0.316***	0.047	−0.006	0.172***	—
	0.003	0.195	< 0.001	0.321	0.903	< 0.001	—

Pearson’s r **p < 0.01, ***p > 0.001.

**Table 7:** Precision of each component in distinguishing ‘Good’ or ‘bad’ play experiences.

	Welch’s t	df	*p*	Cohen’s d
Play feeling inclusion	20.00	290	< 0.001	1.920
	7.35	430	< 0.001	0.686
Imagination	3.63	429	< 0.001	0.345
Physical wildness	−5.10	416	< 0.001	0.478
Something to do	8.42	362	< 0.001	−0.812
Accessibility	7.43	375	< 0.001	0.704
Transgression and silliness	1.12	437	0.263	0.105

Conversely, the remaining scales in the seven-factor model, notably except *silliness and transgression*, give us partial answers to the question of what features some children, more or less strongly call attention to when describing a play experience that fitted their varying notions of what constitutes good or bad play situations.

Interestingly, *silliness and transgression* which could be said to represent the more uncontrolled and carnivalesque elements of play, is neither clearly good or bad. An interpretation could be, that this such behaviours and experiences are thrilling, playful and entertaining to some, but scary, uncertain and disruptive (in a negative sense) to others. This finding highlights the ambiguity of play (as per Sutton-Smith, 1997), both as a phenomenon that is not easily boiled down to a finite set of self-report statements, but also as a practice that is defined by its changing nature, and openness to varied subjective interpretations and preferences—including among school pupils.

In other words, more situational or descriptive factors like wildness, creative imagination, and transgressive boundary crossing occur in enough play memories to constitute factors in the statistical model, but do not seem important to all kids or all play situations. While wildness can easily go too far, it is sometimes experienced as great, and while imagination and creativity are part of many positive moments, they are unnecessary in other play situations, such as rule-based games. These factors need to be understood in a dynamic context, where different kinds of play have different qualities, and where children’s preferences may both be situational *and* vary from child to child. Further, whether these factors constitute a ‘good’ element probably depends on children’s situational or personal preferences, which the present dataset is not equipped to untangle.

Finally, and perhaps most importantly to assessing play difficulties, three factors show the major barriers to good play: Not having anything to do, kills the active feeling of play and social disharmony can either disrupt play or act as a boundary though social exclusion. In contrast to these play problems, some play situations, like big well-organized play activities, seem to afford equal opportunities for entry, trying and getting challenged at your own level which is highly correlated with achieving play feeling.

These insights paint a systematic picture of what good play experiences can look and feel like across over 500 children’s own narratives. They call attention to some of the qualities that educators might want to cultivate, and also the boundaries that may keep children from entering or maintaining a positive play feeling. In addition, it is interesting that factors like transgressive play where you can be cheeky and break the rules, or exclusive play, where a social boundary is maintained against disruptions from others who do not contribute in a positive or competent way, rank is relevant to some children’ interpretations of what makes play good. In short, ‘good’ play, according to children, is not necessarily what adult arbiters might consider ‘nice’ play.

This relationship between factors suggests that well-meaning educators who attempt to force a child into existing play activities may be placing that child in a socially precarious situation, as well as undermining the play order itself. Although some elements seem more universal than others, our findings illustrate that there are several variable dimensions to children’s preferences. We suggest that in order to facilitate inclusive and enjoyable play situations, researchers, designers and professionals may do well in orienting themselves towards such variations, rather than the much more common approach of measuring play as, e.g., developmentally appropriate, pro- or antisocial, instrumentally useful, or in accordance with other norms formulated by adult theories, where the preferences of the children and more subtle developmental opportunities are at risk of being lost.

A main conclusion must be that play situations with an appropriate accessibility threshold allows both entry and experiences of competent participation. This is clearly seen in play that creates a ‘play order’ around, e.g., a creative or performative activity, or large organized games with clear rights and demands, whereas exclusion typically occurs though social misalignment or misaligned expectations, or because play activities do not provide opportunities that are experienced as accessible, appropriate or interesting. Children, however, clearly differ in their play preferences, and the 504 participants in this study thought about many different play experiences when responding. The importance of situations and culture has often been noted (Westby, 2000), suggesting that the factors might differ in other cultural contexts, and that it is worth both allowing children to play freely on their own terms, (nicely and educational or not), and igniting new opportunities for children to develop their play repertoires and experience new modes of participation.

In short, we have identified seven (or in more detail, 22) factors that can act as goal posts or measuring sticks when trying to understand what makes play good or bad in the eyes of children. As a tangible product, we developed the Play Qualities Inventory scales as a tool for both designers, investigators and educators to test out these qualities in new play contexts.

## 9 Limitations

We found that children in the target age group had no problems responding to the questionnaire used in the development process when aided by an adult, and that they easily related exemplary statements from interviews to their own memories of good or bad play. Most of the children intuitively understood the five-point Likert scale and seemed ready to respond themselves after only a few trial items. However, the scales have not been tested without adult supervision, which will be the object of further testing.

In our scale development, we found smaller internal consistency than, e.g., Fehr and Russ (2014) found in their adult-assessed data which ranged in the 0.7–0.9 area. It has been called unsurprising when factor structures for, e.g., playfulness fail to replicate in subsequent data collections (Shen et al., 2014; Staempfli, 2007), and may reflect age variations in the participating children, but instruments based on exploratory studies like the present should either aim at a sufficient level of internal consistency for statistical validation, or be carried forward based on face validity and similar content-based criteria of validity and usefulness—especially for short (sub)scales (Widaman et al., 2011).

The scales of wildness and transgression show smaller and negative correlations with Play feeling, suggesting factors that there are factors which some children experience as negative, but other might enjoy more, or that might be tied with positive experiences in some situations but not others. Transgression and wildness might also be experienced as different “flavors” of play feeling altogether—ones that are less tied to a sense of joy and harmony, but underscores mutability and ambiguity if ongoing play negotiation (Lieberoth, 2008), or factors like challenge and excitement which are often central to the *ludus* of gaming (Sherry et al., 2006) rather than perhaps the *paidia* of free and creative play.

The PQI is not intended as a tool for identifying types of play. However, since each child had reported the nature of the particular play memory they had in mind when responding, it is possible to explore the kinds of play experiences, each subscale relates to. For instance, it could be hypothesized that *imagination* would be higher for play memories related to fantasy play or creative tinkering. Discriminant validity in this regard was especially supported by the finding that *Wildness* was significantly more prominent in play situations categorized as physical- rather than object-, symbolic-, or fantasy-play (as per Whitebread et al., 2017), while imagination scores were higher in fantasy- but not in rule-based play. The scales developed can thus be seen to relate both to separate kinds of ‘good’ or ‘bad’ play based on children’s varying preferences, and we have found evidence to support the Play Feeling scale as a tool for discriminating whether a child found a particular play experience ‘good’.

Finally, our scale development was based on a traditional two-step process going from EFA to CFA. Better *α* and *ω* values could easily have been achieved with more items, as is often the case with short scales (Widaman et al., 2011). Scales were intentionally kept short for the purpose of symmetry, to make the complexity manageable for younger children, and to avoid overfitting at the cost of meaningful content validity.

## 10 Applications and future perspectives

In light of their content and attributes of discriminant validity found in Section 7, the scales *Play Feeling*, *Something To Do* and *Accessibility* can be viewed as *general appraisal instruments,* as they identify features that children consider central to good play. Scales that gauge features related to *imagination*, *wildness*, *transgression* and *social disharmony*, whoever, may be uniquely relevant to certain play experiences only, and thus not applicable to all intended uses. The former may to some degree be used to assess the basic qualit*y* of play, while the latter identify qualit*ies*, of children’s play experiences.

Although cutoffs for reliability statistics are rules of thumb, Monte Carlo simulations have suggested that for large datasets, McDonald’s ω should be >0.65 (Najera, 2019). As can be seen, this was not achieved for all scales, making ‘Play feeling’, ‘Disharmony and Exclusion,’ and ‘Having something to do’ the most internally reliable measures. As the other factors do not apply equally to all play experiences, this suggests that Play Factor scales should be used in relevant contexts where the presence of imagination, wildness, accessibility, and transgression are expected.

In practical use, the large ‘Play feeling’ and ‘Social disharmony’ scales may be especially valid in schools, to evaluate experiences or play landscapes in terms of opportunities for positive participation. In parallel, the full PQI Inventory can map out children‘s daily play experiences using all seven dimensions including elements of specific interest such as imagination which may not be immediately visible, or barriers like accessibility and having something to do, for groups or individuals.

Use scenarios include evaluation of interventions, drawing time series of shifting play experiences over days, or (by asking about ‘good play’ and ‘bad play’ in general) seeking patterns in children’s individual play preferences that might be helpful to their educators. Removing the flexible items (marked with^ in Appendix A), for instance to exclude creation but retain performance in the imagination scale, will ensure contextual validity.

The latter part of our analysis focused on operationalizations of the seven-component model into self-report scales, while the more varied and exploratory first extraction is included to show the variety of factors at play in what children experience as good, bad, or noteworthy features of common play experiences. While both overlaps and differences in adult interpretations of play have been found in cultural studies (Li et al., 1995; Mills, 1988), no studies at the scale of the present investigation have compared children’s views of what constitutes ‘good’ or ‘bad’ play.

There are several issues with fitting CFA on the same dataset, so the next logical step will be to test reliability by collecting new sets of responses (Fokkema and Greiff, 2017). A tantalizing prospect beyond testing the *‘Play Qualities Inventory’ (PQI)* scales outside the Danish context, would therefore be to apply similar processes to other cultural contexts, in order to ask the big question: Are play qualities experienced differently based on norms and upbringing, and would replications lead to other sets of factors in China, South America, or the Middle East? Our expectation would be yes. It is likely that a multitude of factors exist—but some may indeed be general to kids everywhere.

## Data Availability

The raw data supporting the conclusions of this article will be made available by the authors, without undue reservation.
