# Supplementary material for: Seven core qualities of good vs. bad play? A principal component analysis of 504 children’s play memories and development of a Play Qualities Inventory
Source: Front Psychol. 2026 Mar 27;17:1690952. doi: 10.3389/fpsyg.2026.1690952 (PMC13066295; doi:10.3389/fpsyg.2026.1690952)
Supplement: Supplementary file 1 [file Table_1.docx]

# Appendix A:

# *Play Qualities Inventory* (PQI) items

*PQI scales and items may be used, reproduced and modified together or individually under the Creative Commons CC-BY-4.0 license, with attribution to the original authors, and reference to the original materials and published paper by Author et al. 202X from Frontiers on Psychology.*

|  |
| --- |
| \| **Scale: Play feeling** \| \| \| \| \| \| \| \| \| \| \| --- \| --- \| --- \| --- \| --- \| --- \| --- \| --- \| --- \| --- \| \|  \| \| \| \| \| \| **if item dropped** \| \| \| \| \|  \| \| **mean** \| \| **sd** \| \| **Cronbach's α** \| \| **McDonald's ω** \| \| \| It was nice \|  \| 4.03 \|  \| 1.36 \|  \| 0.833 \|  \| 0.840 \|  \| \| I could get a smile on my mouth \|  \| 4.07 \|  \| 1.32 \|  \| 0.851 \|  \| 0.859 \|  \| \| It was annoying ᵃ \|  \| 3.57 \|  \| 1.62 \|  \| 0.846 \|  \| 0.860 \|  \| \| I couldn't be bothered (to participate) ᵃ \|  \| 3.88 \|  \| 1.48 \|  \| 0.867 \|  \| 0.877 \|  \| \| I got sad inside ᵃ \|  \| 4.09 \|  \| 1.43 \|  \| 0.877 \|  \| 0.886 \|  \| \| You are happy \|  \| 4.08 \|  \| 1.29 \|  \| 0.835 \|  \| 0.840 \|  \| \| ᵃ reverse scored item  ^ Expanded scale social play items \| \| \| \| \| \| \| \| \| \| \|  \| \| \| \| \| \| \| \| \| \| |

| \| **Expanded scale: Social play feeling** \| \| \| \| \| \| \| \| \| \| \| --- \| --- \| --- \| --- \| --- \| --- \| --- \| --- \| --- \| --- \| \|  \| \| \| \| \| \| **if item dropped** \| \| \| \| \|  \| \| **mean** \| \| **sd** \| \| **Cronbach's α** \| \| **McDonald's ω** \| \| \| It was nice/cosy \|  \| 4.03 \|  \| 1.36 \|  \| 0.868 \|  \| 0.875 \|  \| \| I could get a smile on my mouth \|  \| 4.07 \|  \| 1.32 \|  \| 0.877 \|  \| 0.885 \|  \| \| It was annoying ᵃ \|  \| 3.57 \|  \| 1.62 \|  \| 0.872 \|  \| 0.883 \|  \| \| I couldn't be bothered (to participate) ᵃ \|  \| 3.88 \|  \| 1.48 \|  \| 0.883 \|  \| 0.893 \|  \| \| I got sad inside ᵃ \|  \| 4.09 \|  \| 1.43 \|  \| 0.886 \|  \| 0.897 \|  \| \| You are happy \|  \| 4.08 \|  \| 1.29 \|  \| 0.867 \|  \| 0.872 \|  \| \| Everyone had fun together^ \|  \| 3.83 \|  \| 1.41 \|  \| 0.873 \|  \| 0.881 \|  \| \| We ended up being unfriendly ᵃ^ \|  \| 3.43 \|  \| 1.67 \|  \| 0.894 \|  \| 0.900 \|  \| \| ᵃ reverse scored item  ^ Expanded scale social play items \| \| \| \| \| \| \| \| \| \| \|  \| \| \| \| \| \| \| \| \| \| |
| --- | --- | --- | --- | --- | --- | --- | --- | --- | --- | --- | --- | --- | --- | --- | --- | --- | --- | --- | --- | --- | --- | --- | --- | --- | --- | --- | --- | --- | --- | --- | --- | --- | --- | --- | --- | --- | --- | --- | --- | --- | --- | --- | --- | --- | --- | --- | --- | --- | --- | --- | --- | --- | --- | --- | --- | --- | --- | --- | --- | --- | --- | --- | --- | --- | --- | --- | --- | --- | --- | --- | --- | --- | --- | --- | --- | --- | --- | --- | --- | --- | --- | --- | --- | --- | --- | --- | --- | --- | --- | --- | --- | --- | --- | --- | --- | --- | --- | --- | --- | --- | --- | --- | --- | --- | --- | --- | --- | --- | --- | --- | --- | --- | --- | --- | --- | --- | --- | --- | --- | --- | --- | --- | --- | --- | --- | --- | --- | --- | --- | --- |

| **Inclusion** | | | | | | | | | | |
| --- | --- | --- | --- | --- | --- | --- | --- | --- | --- | --- |
|  | | | | | | **if item dropped** | | | | |
|  | | **mean** | | **sd** | | **Cronbach's α** | | **McDonald's ω** | |  |
| There was someone, who made me sad ᵃ |  | 1.89 |  | 1.41 |  | 0.699 |  | 0.708 |  |  |
| I couldn't find anyone to be with ᵃ |  | 1.81 |  | 1.42 |  | 0.662 |  | 0.684 |  |  |
| I was being kept out ᵃ |  | 1.62 |  | 1.24 |  | 0.594 |  | 0.604 |  |  |
| There was somebody, who wouldn't let me join in ᵃ |  | 1.67 |  | 1.29 |  | 0.620 |  | 0.634 |  |  |

ᵃ reverse scored item

| **Expanded scale: Inclusion and coherence** | | | | | | | | | |
| --- | --- | --- | --- | --- | --- | --- | --- | --- | --- |
|  | | | | | | **if item dropped** | | | |
|  | | **mean** | | **sd** | | **Cronbach's α** | | **McDonald's ω** | |
| There was someone, who made me sad ᵃ |  | 1.90 |  | 1.41 |  | 0.654 |  | 0.675 |  |
| I couldn't find anyone to be with ᵃ |  | 1.81 |  | 1.42 |  | 0.638 |  | 0.657 |  |
| I was being kept out ᵃ |  | 1.62 |  | 1.24 |  | 0.628 |  | 0.633 |  |
| There was somebody, who wouldn't let me join in ᵃ |  | 1.67 |  | 1.30 |  | 0.635 |  | 0.643 |  |
| Someone tried to push their way into our game ᵃ^ |  | 2.10 |  | 1.50 |  | 0.688 |  | 0.711 |  |
| The others interrupted ᵃ ^ |  | 2.42 |  | 1.63 |  | 0.667 |  | 0.693 |  |
| ᵃ reverse scored item  ^ Expanded scale play coherence items |  |  |  |  |  |  |  |  |  |

| **Imagination** | | | | | | | | | | |
| --- | --- | --- | --- | --- | --- | --- | --- | --- | --- | --- |
|  | | | | | | **if item dropped** | | | | |
|  | | **mean** | | **sd** | | **Cronbach's α** | | **McDonald's ω** | |  |
| I came up with something / had ideas |  | 2.902 |  | 1.667 |  | 0.484 |  | 0.490 |  |  |
| You could use your imagination |  | 3.306 |  | 1.673 |  | 0.512 |  | 0.524 |  |  |
| I played that it was real |  | 2.790 |  | 1.697 |  | 0.548 |  | 0.550 |  |  |
| I tried something new |  | 3.027 |  | 1.626 |  | 0.538 |  | 0.538 |  |  |

| **Expanded scale: Imagination and performance/creativity** | | | | | | | | | |
| --- | --- | --- | --- | --- | --- | --- | --- | --- | --- |
|  | | | | | | **if item dropped** | | | |
|  | | **mean** | | **sd** | | **Cronbach's α** | | **McDonald's ω** | |
| I came up with ideas |  | 2.92 |  | 1.66 |  | 0.608 |  | 0.615 |  |
| You could use your imagination |  | 3.29 |  | 1.67 |  | 0.643 |  | 0.653 |  |
| I played that it was real |  | 2.81 |  | 1.69 |  | 0.668 |  | 0.673 |  |
| I tried something new |  | 3.05 |  | 1.62 |  | 0.660 |  | 0.667 |  |
| I made/did something, I could show to others^ |  | 2.24 |  | 1.53 |  | 0.631 |  | 0.634 |  |
| I made/did something I could keep after^ |  | 2.24 |  | 1.53 |  | 0.644 |  | 0.648 |  |
| ^ Expanded scale play coherence items. Wording can be adapted depending on the nature of play, i.e. performance versus arts/crafts | | | | | | | | | |

| **Physical wildness** | | | | | | | | | | | | | | | | | | | |  |  |
| --- | --- | --- | --- | --- | --- | --- | --- | --- | --- | --- | --- | --- | --- | --- | --- | --- | --- | --- | --- | --- | --- |
|  | | | | | | | | | | | | | **if item dropped** | | | | | | |  |  |
|  | | | | | | | **mean** | | | | **sd** | | **Cronbach's α** | | | | **McDonald's ω** | | |  |  |
| It was a wild game | | | | | |  | 2.63 | |  | | 1.59 |  | 0.491 | | |  | 0.532 |  | |  |  |
| It was tough | | | | | |  | 2.95 | |  | | 1.67 |  | 0.498 | | |  | 0.545 |  | |  |  |
| It got too rough | | | | | |  | 2.20 | |  | | 1.55 |  | 0.514 | | |  | 0.536 |  | |  |  |
| You came to relax ᵃ | | | | | |  | 3.31 | |  | | 1.57 |  | 0.660 | | |  | 0.663 |  | |  |  |
| ᵃ reverse scored item | | | | | | | | | | | | | | | | | | | | | |
|  | | | | | | | | | | | | | | | | | | | |  |  |
| **Something to do** | | | | | | | | | | | | | | | | | | | |  |  |
|  | | | | | | | | | **if item dropped** | | | | | | | | | | |  |  |
|  | | **mean** | | **sd** | | | | | **Cronbach's α** | | | | | **McDonald's ω** | | | | | |  |  |
| I just looked on ᵃ |  | 1.68 |  | 1.30 | | |  | | 0.616 | | | |  | 0.627 | | | |  | |  |  |
| I didn't have anything to do ᵃ |  | 1.88 |  | 1.37 | | |  | | 0.599 | | | |  | 0.611 | | | |  | |  |  |
| I didn't do anything in particular ᵃ |  | 2.37 |  | 1.49 | | |  | | 0.672 | | | |  | 0.679 | | | |  | |  |  |
| I never got to do the most fun things ᵃ |  | 2.12 |  | 1.51 | | |  | | 0.660 | | | |  | 0.672 | | | |  | |  |  |
| ᵃ reverse scored items | | | | | | | | | | | | | | | | | | | | | |
|  | | | | | | | | | | | | | | | | | | | |  |  |

|  |
| --- |

| **Subscale: Accessibility** | | | | | | | | | |
| --- | --- | --- | --- | --- | --- | --- | --- | --- | --- |
|  | | | | | | **if item dropped** | | | |
|  | | **mean** | | **sd** | | **Cronbach's α** | | **McDonald's ω** | |
| You could practice and get better |  | 4.02 |  | 1.39 |  | 0.608 |  | 0.669 |  |
| Everyone was allowed to join in |  | 4.07 |  | 1.41 |  | 0.458 |  | 0.476 |  |
| Everyone was allowed to try |  | 4.01 |  | 1.44 |  | 0.374 |  | 0.383 |  |
| I was good at it |  | 4.14 |  | 1.22 |  | 0.539 |  | 0.600 |  |
|  | | | | | | | | | |

| **Subscale: Transgression and silliness** | | | | | | | | | |
| --- | --- | --- | --- | --- | --- | --- | --- | --- | --- |
|  | | | | | | **if item dropped** | | | |
|  | | **mean** | | **Sd** | | **Cronbach's α** | | **McDonald's ω** | |
| You could get up to no good together |  | 3.17 |  | 1.57 |  | 0.466 |  | 0.474 |  |
| I could be cheeky |  | 3.07 |  | 1.59 |  | 0.516 |  | 0.526 |  |
| It was silly |  | 3.13 |  | 1.61 |  | 0.548 |  | 0.588 |  |
| You could change the game along the way |  | 2.74 |  | 1.61 |  | 0.632 |  | 0.648 |  |
|  | | | | | | | | | |

All components and items translated from Danish child statements discussed in Author et al. 202X, and re-worded appendices B & C.

The *PQI* was developed with Danish primary school pupils, age 5-11, for answers on 4 point Likert scale with adult supervision/support for younger children.

The authors welcome correspondence and request to be notified on any data collection or publications wholly or partially based on these materials,

“These are some thins other kids your age have said about their good and bad play experiences. See if they fit [Play situation – e.g. “the free play time after school yesterday” “when you play with the adults here”, etc.]

[Item] Was this true or not so true of [your play experience]?:

Completely true (4), a little true (3) , a little false (2), Completely false (1).
